# Sex Differences in Hospital-Acquired Pneumonia among Patients with Type 2 Diabetes Mellitus Patients: Retrospective Cohort Study using Hospital Discharge Data in Spain (2016–2019)

**DOI:** 10.3390/ijerph182312645

**Published:** 2021-11-30

**Authors:** Ana Lopez-de-Andres, Marta Lopez-Herranz, Valentin Hernandez-Barrera, Javier de-Miguel-Diez, Jose M. de-Miguel-Yanes, David Carabantes-Alarcon, Romana Albaladejo-Vicente, Rosa Villanueva-Orbaiz, Rodrigo Jimenez-Garcia

**Affiliations:** 1 Department of Public Health & Maternal and Child Health, Faculty of Medicine, Universidad Complutense de Madrid, 28040 Madrid, Spain; anailo04@ucm.es (A.L.-d.-A.); dcaraban@ucm.es (D.C.-A.); ralbadal@med.ucm.es (R.A.-V.); mrvillan@med.ucm.es (R.V.-O.); rodrijim@ucm.es (R.J.-G.); 2 Physiotherapy and Podology Nursing Department, Faculty of Nursing, Universidad Complutense de Madrid, 28040 Madrid, Spain; 3 Preventive Medicine and Public Health Teaching and Research Unit, Health Sciences Faculty, Universidad Rey Juan Carlos, 28922 Madrid, Spain; valentin.hernandez@urjc.es; 4 Respiratory Department, Hospital General Universitario Gregorio Marañón, Instituto de Investigación Sanitaria Gregorio Marañón (IiSGM), Universidad Complutense de Madrid, 28040 Madrid, Spain; javier.miguel@salud.madrid.org; 5 Internal Medicine Department, Hospital General Universitario Gregorio Marañón, Instituto de Investigación Sanitaria Gregorio Marañón (IiSGM), Universidad Complutense de Madrid, 28040 Madrid, Spain; josemaria.demiguel@salud.madrid.org

**Keywords:** hospital-acquired pneumonia, ventilator-associated pneumonia, type 2 diabetes mellitus, hospitalizations, sex differences, outcomes

## Abstract

(1) Background: To analyze the incidence, clinical characteristics, use of procedures, and in-hospital outcomes in patients who developed pneumonia during their hospital admission according to sex and to the presence of type 2 diabetes mellitus (T2DM). (2) Methods: Retrospective cohort study using data from the Spanish National Hospital Discharge Database. Hospital-acquired pneumonia (HAP) was classed as non-ventilator HAP and ventilator-associated pneumonia (VAP). Separate analyses were performed for men and women with and without T2DM. Population subgroups were compared using propensity score matching. (3) Results: HAP was identified in 38,814 patients (24.07% with T2DM). The adjusted incidence of HAP was higher in patients with T2DM (both sexes) (IRR 1.28; 95% CI 1.25–1.31). The incidence of HAP was higher in men with T2DM than in women with T2DM (adjusted-IR 1.47; 95% CI 1.41–1.53). The incidence of HAP among T2DM patients increased over time. In-hospital mortality (IHM) was around 28% irrespective of T2DM status and sex. After adjusting for confounders and sex, VAP was associated to higher IHM among patients with T2DM (OR 2.09; 95% CI 1.7–2.57). (4) Conclusions: T2DM is associated with a higher risk of HAP, whose incidence increased over time. Men with T2DM have an almost 50% higher risk of HAP than women with T2DM. The probability of dying in the hospital was not associated with sex or T2DM.

## 1. Introduction

Hospital-acquired pneumonia (HAP) is the most common nosocomial infection. Its incidence increases over time and varies considerably with age and comorbidities [1]. HAP more commonly occurs in non-ventilated patients [2,3]. In their analysis of the US National Inpatient Sample dataset, Giuliano et al. [4] found that non-ventilator hospital-acquired pneumonia (NV-HAP) led to an increase in costs, length of stay, and mortality in hospitalized patients.

Ventilator-associated pneumonia (VAP), a subgroup of HAP, is the most common infection among patients undergoing mechanical ventilation. It has been associated with increased mortality (approaching 50%), increased morbidity, length of intensive care unit (ICU) stay, length of hospital stay (LOHS), and time on mechanical ventilation [5]. Although both NV-HAP and VAP generate relevant clinical and economic burdens, most reports focus on VAP [6].

People with type 2 diabetes mellitus (T2DM) are at a greater risk of infection and have poorer outcomes than those without diabetes [7]. Nonetheless, the relationship between HAP and T2DM has not been thoroughly analyzed in the literature. Although T2DM was recently reported to be an independent predictor of VAP [8], there are very few studies on NV-HAP and T2DM [9].

The incidence and outcomes of patients with HAP vary according to sex. Some authors report that incidence might be higher among men [10,11,12], although others conclude that female sex is a major risk factor for mortality after HAP [13]. Consequently, reported data are contradictory [14]. Few data are available on the results of HAP among men and women with diabetes.

Given these inconclusive findings, our study was based on national administrative data taken over a four-year period to compare the incidence, clinical characteristics, use of therapeutic procedures, and in-hospital outcomes in patients who developed HAP during hospital admission according to T2DM and sex. We used propensity score matching (PSM) to compare outcomes after HAP between men and women with and without T2DM and between men and women with T2DM. Finally, we identified the variables associated with in-hospital mortality (IHM) for patients with T2DM according to sex.

## 2. Materials and Methods

### 2.1. Study Design, Study Population, and Data Assessment

We performed a cohort study based on hospital discharge reports collected through the Hospital Discharge Records of the Spanish National Health System (RAE-CMBD, Registro de Actividad de Atención Especializada-Conjunto Mínimo Básico de Datos (Register of Specialized Care–Basic Minimum Database)) for the period running from 1 January 2016 to 31 December 2019. The discharge records are coded based on the International Classification of Disease, Tenth Revision (ICD-10). Details on the RAE-CMBD are available online [15].

The study population comprised patients aged ≥18 years whose primary diagnosis was HAP based on the specific diagnosis of NV-HAP or VAP, which was assigned according to the ICD-10 codes recorded in the discharge records, as indicated in Table S1.

We classified the study population according to sex and to the presence of T2DM. Patients with a diagnosis code for T2DM (E11.x) in any field were classified as having T2DM. Patients with a code for type 1 diabetes mellitus (E10.x) in any field were excluded. 

The main study variables were trends in the incidence of HAP, NV-HAP, and VAP in patients with and without T2DM and the IHM and length of hospital stay (LOHS) of patients with HAP. We also analyzed comorbidities and therapeutic procedures in in-patients with HAP.

Incidence rates were based on the Spanish population hospitalized with and without T2DM classified by age group and sex according to the RAE-CMBD [16].

Comorbidity was quantified using the Charlson Comorbidity Index (CCI) calculated based on ICD-10 codes, as described elsewhere [17,18]. 

The RAE-CMBD includes a variable with the diagnosis-related groups categorized as Medical/Surgical/Other. This approach was used to identify patients who underwent surgery during admission [15].

The procedures studied were fiberoptic bronchoscopy, computerized axial tomography of the thorax, dialysis, and oxygen prior to hospitalization (see ICD-10 codes in Table S1).

As for pathogens in patients with pneumonia, we only included those confirmed in the laboratory, as follows: *Aspergillus*, *Candidiasis*, *Escherichia coli*, *Haemophilus influenzae*, *Klebsiella pneumoniae*, *Legionella*, non-specified *Streptococcus*, other Gram-negative bacteria, *Pseudomonas aeruginosa*, *Staphylococcus aureus*, *Streptococcus pneumonia*, influenza virus, and other viruses (see ICD-10 codes in Table S1).

We used PSM to create subpopulations that were comparable based on their baseline conditions [19]. We performed three PSM analyses: women with T2DM and non-T2DM women, men with T2DM and non-T2DM men, and T2DM men and T2DM women. 

To conduct the propensity score matching, we followed the recommendations of Austin and Luellen et al. [19,20]. First, we applied logistic regression computing to estimate propensity scores for each patient. The variables included in the model were age, sex, and comorbid conditions present at admission [9]. Second, we balanced the nonequivalent groups with the nearest neighbor matching method. Nearest neighbor matching selects for matching to a given subject of the exposed population a subject of the unexposed population whose propensity score is closest to that of the exposed population [19]. If multiple subjects have propensity scores that are equally close to that of the exposed subject, one of these unexposed subjects is selected at random. It is important to note that no restrictions are placed upon the maximum acceptable difference between the propensity scores of two matched subjects [19]. Third, to validate the model, we subject each continuous covariate to a two-way ANOVA and assess the interaction term. When the interaction term is non-significant, we drop it from the model and rerun the ANOVA to more accurately assess the main effect of the variable. We used logistic regression in a similar two-step procedure for assessing the balance of dichotomous categorical variables [20]. 

### 2.2. Statistical Analysis

We obtained the incidence of HAP per patient hospitalized with and without T2DM for each of the four years analyzed. Incidence was analyzed using Poisson regression models adjusted for age and sex as applicable. The RAE-CMBD does not include the date for each of the diagnosis codified [15]. This database only includes the date of admission and discharge, so we do not have the time since the patient was admitted to the hospital until the HAP was diagnosed. Therefore, the length of stay until the diagnosis of HAP could not be included in the Poisson regression analysis. Incidence rate ratios (IRR) with their 95% confidence intervals (95% CI) were reported. 

The descriptive statistical analysis included mean and standard deviation (SD) or median and interquartile range (IQR) for continuous variables and frequency and percentage for categorical variables.

Continuous variables were compared using the t test or Mann–Whitney test. Categorical variables were compared using the chi-square test.

We conducted multivariable logistic regression analysis to determine which variables were independently associated with IHM. Separate models were built for men and women and according to T2DM status. Finally, we analyzed the effect of sex using the entire database of patients with T2DM. The results are shown as odds ratios (ORs) with their 95% CIs.

IHM could have been analyzed using Poisson regression models, as has been reported before [21]. This method is considered the optimal option for count variables such as LOHS [21]. However, we preferred logistic regression because for binary variables, such as IHM, these models seem to provide more accurate estimates [22,23,24]. Furthermore, according to the literature, Poisson regression is very useful for rare events (<5%), whereas logistic regression provides better multivariable adjustment when the event is more frequent (>5%) [22]. In our study, the IHM was around 30%. The validity of logistic regression to assess factors associated to IHM has been reported previously, even when compared to more recent statistical methods such as machine learning methods [23,24].

The statistical analysis and PSM were conducted using Stata version 14 (Stata, College Station, TX, USA), and significance was set at *p* < 0.05 (2-sided). 

### 2.3. Ethics

The RAE-CMBD is owned by the Spanish Ministry of Health and can be accessed upon request [25]. According to Spanish legislation, as this registry is anonymous, neither individual written consent from the patients or ethics committee approval is required.

## 3. Results

A total of 38,814 patients (65.39% men and 34.61% women) aged ≥18 years were hospitalized with a primary diagnosis of HAP in Spain during the period 2016–2019. A diagnosis of NV-HAP was identified in 33,588 patients (86.53%) and a diagnosis of VAP was identified in 5226 patients (13.47%).

T2DM was diagnosed in 9343 patients (24.07%) with HAP. T2DM was more prevalent in men than in women with HAP (24.26% vs. 23.71%; *p* < 0.001).

### 3.1. Incidence of HAP during Hospital Admission According to T2DM Status

In patients with T2DM, the incidence of HAP increased significantly from 279.43 cases per 100,000 persons hospitalized with T2DM in 2016 to 296.47 in 2019 (*p* < 0.001) (Table 1). The incidence of NV-HAP and VAP also increased significantly during the study period in patients with and without T2DM and was significantly higher in patients with T2DM than in non-T2DM persons for both types of pneumonia and for all the years analyzed (*p* < 0.001). However, the incidence of VAP was significantly higher in non-T2DM persons for the years 2017 (*p* < 0.001) and 2019 (*p* = 0.04). 

Using the Poisson regression model, we found that the incidence of HAP was 1.28-fold higher among patients with T2DM than among non-T2DM patients (IRR 1.28; 95% CI 1.25–1.31).

The mean CCI increased significantly in persons with and without T2DM. LOHS was around 22 and 24 days in patients with and without T2DM, respectively. IHM was around 28% for all years and irrespective of T2DM status.

The frequency of the influenza virus increased significantly over time in patients with T2DM (*p* = 0.033); however, it decreased in patients without T2DM (*p* < 0.001). The frequency of *Streptococcus pneumoniae* increased significantly from 2.12% in 2016 to 3.01% in 2019 (*p* = 0.003), although only in patients without T2DM (Table S2).

### 3.2. Clinical Characteristics and Hospital Outcomes for Women and Men Who Developed HAP during Admission by T2DM Status

Table 2 shows the incidence, clinical characteristics, therapeutic procedures, and hospital outcomes before and after PSM for women with HAP according to the presence of T2DM.

The incidence was significantly higher in women with T2DM than in those without T2DM (240.15 cases per 100,000 women hospitalized with T2DM vs. 140.65 cases per 100,000 women hospitalized without T2DM, *p* < 0.001). The incidence of NV-HAP was also significantly higher in women with T2DM (all *p* < 0.001). No differences were found in the incidence of VAP between women with and without T2DM.

Mean age was significantly higher in women with T2DM (77.65; SD = 11.17 years old) than in those who did not have T2DM (70.77; SD = 17.06 years old), and women with T2DM also had a higher mean CCI and more specific chronic conditions, namely, a higher prevalence of myocardial infarction, congestive heart failure, peripheral vascular disease, cerebrovascular disease, dementia, and kidney disease. 

During hospitalization, women with T2DM underwent fiberoptic bronchoscopy and surgery significantly less often than women without T2DM (both *p* < 0.001); however, women with T2DM more frequently received oxygen prior to hospitalization (3.42% vs. 2.46%; *p* = 0.003). The mean LOHS was significantly lower in women with T2DM than in non-T2DM women (21 days vs. 23 days). The crude IHM was 30.39% for women with T2DM and 27.16% for non-T2DM women (*p* < 0.001).

After PSM, surgery continued to be less frequent among T2DM women (36.58% vs. 40.97%; *p* < 0.001). However, IHM was 30% in both women with and women without T2DM.

Table 3 shows the incidence, clinical characteristics, therapeutic procedures, and hospital outcomes before and after PSM for men with and without T2DM hospitalized with HAP. 

The crude incidence of HAP was significantly higher in men with T2DM than in non-diabetic men (336.68 cases per 100,000 men hospitalized with T2DM vs. 326.62 cases per 100,000 men hospitalized without T2DM; *p* = 0.038). The incidence of NV-HAP was also significantly higher in men with T2DM, although the incidence of VAP was lower in men with T2DM (all *p* < 0.001). 

Before performing PSM, we found significant differences in age distribution and comorbidities (CCI) between men with and without T2DM, as was the case in women.

Men with T2DM less frequently underwent fiberoptic bronchoscopy, computerized axial tomography of the thorax, and surgery (all *p* < 0.001); however, the prevalence of dialysis and oxygen prior to hospitalization was significantly higher in men with T2DM. LOHS was lower in diabetic men (22 days vs. 25 days; *p* < 0.001). T2DM men had higher crude IHM than men without T2DM (30.29% vs. 28.77%, *p* = 0.023).

After PSM, we found that among diabetic men, computerized axial tomography of the thorax and surgery continued to be significantly less frequent than in non-T2DM men (6.95% vs. 8.36% and 44.56% vs. 48.16%, respectively). LOHS in men with T2DM continued to be lower (*p* = 0.014), although IHM was around 30% in men with and without T2DM.

### 3.3. Incidence, Clinical Characteristics, and Hospital Outcomes for Diabetic Patients who Developed HAP during Hospital Admission According to Sex

Incidence was significantly higher in men than in women with T2DM (Table 4). The results of the Poisson regression model showed that the overall incidence of HAP during 2016–2019 was 1.47-fold higher in men than in women (IRR 1.47; 95% CI 1.41–1.53).

A comparison of T2DM men with T2DM women revealed that men were younger (73.8 ± 10.75 years vs. 77.65 ± 11.17 years; *p* < 0.001), with a higher mean CCI (1.8 ± 1.14 vs. 1.55 ± 1.07). Men also more frequently had myocardial infarction, peripheral vascular disease, COPD, liver disease (mild and moderate/severe), and cancer/metastatic cancer. However, congestive heart failure, dementia, and rheumatoid arthritis were more prevalent in women than in men. 

Before PSM, men more frequently underwent fiberoptic bronchoscopy (2.35% vs. 1.55%; *p* = 0.006) and dialysis (8.23% vs. 5.68%; *p* < 0.001). Furthermore, the prevalence of surgery was higher in men (44.56% vs. 36.58%; *p* < 0.001).

After PSM, no significant differences were found between men and women regarding therapeutic procedures, hospital stay, and IHM.

As for the isolation of pathogens, the prevalence of *Escherichia coli*, *Klebsiella pneumoniae*, non-specified *Streptococcus*, and other Gram-negative bacteria after PSM was significantly higher in men than in women (Table S3).

### 3.4. Variables Associated with IHM in Diabetic Men and Women Who Developed HAP during Hospital Admission: Multivariable Analysis

As can been seen in Table 5, the IHM increased with age, myocardial infarction, congestive heart failure, peripheral vascular disease, cerebrovascular disease, and cancer/metastatic cancer in both men and women with T2DM. However, dementia (OR, 1.29 (95% CI, 1.01–1.67)) and mild moderate/severe liver disease (OR, 1.28 (95% CI 1.06–1.53)) were associated with IHM in men.

In both men and women, surgery reduced IHM (OR, 0.8 (95% CI 0.71–0.91)). However, the need for fiberoptic bronchoscopy and dialysis during admission increased IHM in T2DM patients irrespective of sex (OR, 2.01 (95% CI, 1.31–3.11) and OR, 2.15 (95% CI 1.72–2.67), respectively).

When possible, confounders were controlled for, and VAP increased two-fold IHM in both men and women (OR, 2.09 (95% CI, 1.7–2.57)). No association was found with year of admission in either men or women. Finally, as in the PSM analysis, sex was not associated with IHM (male sex: OR, 1.03 (95% CI, 1.7–2.57)).

## 4. Discussion

This nationwide registry and population-based observational cohort study showed that incidence rates of HAP in patients with T2DM were higher than in those without T2DM for all the years analyzed. However, this was due to NV-HAP, since the incidence of VAP was higher in subjects without T2DM in the years 2017 and 2019. Incidence was significantly higher in men with T2DM than in women. In the fully adjusted model, among T2DM patients who suffered an HAP, those with VAP had an OR of 2.09 for IHM.

Our database showed that the frequency of HAP increased significantly from 2016 to 2019 irrespective of T2DM status. This trend is not consistent with data from other population-based studies [3,26], which is probably because of methodological differences in the calculation of HAP rates. 

The higher incidence of HAP, especially NV-HAP, in patients with T2DM irrespective of sex is consistent with data reported elsewhere [27]. The greater susceptibility of T2DM patients to HAP may be due to clinical characteristics, such as poor glycemic control [28]. However, in our study, it is striking that the incidence of VAP was lower in men with T2DM than in those without T2DM, thus contradicting several earlier studies that identified T2DM as an independent risk factor for the development of VAP [29]. Similarly, Tsakiridou et al. [30] found no significant relationship between diabetes and VAP and concluded that a combination of factors (micro-aspirations, increased severity of illness, and duration of mechanical ventilation) might increase the frequency of VAP. In our study, diabetic patients were older and had more comorbid factors than non-diabetic patients. In any case, it is important to remember that only patients admitted to an ICU and connected to a ventilator can acquire VAP. Based on admission criteria, T2DM patients are probably admitted to the ICU in a lower proportion than non-diabetic patients owing to their more advanced age and poorer overall health status [31].

As expected, the incidence rates of HAP, NV-HAP, and VAP were higher in T2DM men than in T2DM women. These results agree with data from general populations [12,26,32], where men have been identified as having a higher incidence of VAP in all age groups and a poorer prognosis [33]. Furthermore, in the United States, a cohort study conducted during 2013–2017 found that male sex was associated with an increased risk of NV-HAP (female sex: HR 0.77; 95% CI 0.59–1.00) [34].

Despite improved preventive measures, antimicrobial therapy, and supportive care [33], HAP continues to be a major cause of morbidity and mortality and has been associated with significantly high mortality [35,36]. In our study, IHM was similar in T2DM patients and in non-T2DM patients, with no significant change over time. Furthermore, sex was not associated with IHM, thus confirming previous findings suggesting that sex may influence the incidence of infection but not its severity [37].

Our findings agree with those reported elsewhere, namely, that patients with diabetes have a worse risk profile than patients without diabetes [7]. Again, consistent with other authors and as expected, we found the risk factors for IHM to be older age, myocardial infarction, congestive heart failure, peripheral vascular disease, cerebrovascular disease, and cancer/metastatic cancer [9]. Fiberoptic bronchoscopy and dialysis were significantly more frequent in the T2DM patients who died than in those who survived.

As we expected, previous surgery was associated with reduced mortality in patients with HAP. In our opinion, the explanation for this association is that older patients with T2DM and poorer health status are less likely to undergo surgery.

In the current study, we showed that VAP increased the OR of dying in both men and women with T2DM. Previous studies also reported that T2DM is associated with increased mortality rates in VAP patients [38,39]. The several factors that could explain the higher rates of mortality in T2DM patients with VAP include older age and more frequent comorbidity, which increase vulnerability to severe complications, such as organ failure, and lead to earlier and more frequent death [31]. Furthermore, hyperglycemia in non-diabetic persons had a significantly negative effect on patient survival [40]. However, we did not pursue this hypothesis, which could be the subject of a future study.

The strength of our study lies in its large sample size (>38,814 episodes of HAP, 24.07% with T2DM), coverage of an entire country (>95% of all hospital admissions), and standardized methodology (extensively used for research in Spain, as well as the reliability of diabetes and HAP coding in the RAE-CMBD [9,12,26,31]). Nevertheless, our study is subject to a series of limitations. First, our data were obtained from an administrative database supported by the information that physicians recorded in the discharge report; therefore, the database lacks information on clinical characteristics, glycemic control, medical treatments, and duration of T2DM. Furthermore, data other than those included in the ICD-10 code for duration of ventilatory support, days in the ICU, vaccinations, and severity of the respiratory disease were not available. Second, although PSM went some way to mitigating differences in baseline characteristics and clinical variables, complete elimination of residual confounding is difficult in observational studies. Third, anonymity precludes the extraction of specific data that may affect the results (i.e., people who moved from one hospital to another could appear twice). Fourth, survival analysis could not be conducted because, as commented before, we lack the information on the time from hospital admission to HAP diagnosis as well as the time from HAP diagnosis to death or hospital discharge.

## 5. Conclusions

In conclusion, T2DM is associated with a higher incidence of HAP, which is associated mainly with NV-HAP but not with VAP. This incidence continues to increase. When stratifying by sex, we found that the difference was based mainly on the preponderance of male sex. IHM was similar in T2DM patients and in non-T2DM patients, with no significant change over time. In addition, sex was not associated with IHM, suggesting that it may influence the incidence but not the prognosis of HAP. Our findings should be taken into consideration when planning actions to improve the treatment and care provided to T2DM patients who develop HAP during hospital admission. Research efforts should focus on identifying and eliminating these sex-related disparities in our health system.

## Figures and Tables

**Table 1:** Incidence, clinical characteristics, and in-hospital outcomes of patients who developed hospital-acquired pneumonia (HAP) in Spain from 2016 to 2019 according to the presence of T2DM.

Variables	Presence of T2DM	2016	2017	2018	2019	*p*-Value
*N* (incidence of HAP per 100,000 subjects hospitalized)	T2DM	1988 (279.43)	2197 (279.61)	2654 (326.25)	2504 (296.47)	<0.001
	No T2DM	6596 (202.36)	7250 (216.36)	7719 (236.14)	7906 (240.17)	<0.001
*N* (incidence of NV-HAP per 100,000 subjects hospitalized)	T2DM	1789 (251.46)	2030 (258.35)	2397 (294.66)	2219 (262.72)	<0.001
	No T2DM	5696 (147.75)	6223 (185.71)	6598 (201.85)	6636 (201.59)	<0.001
*N* (incidence of VAP per 100,000 subjects hospitalized)	T2DM	199 (27.97)	167 (21.25)	257 (31.59)	285 (33.74)	<0.001
	No T2DM	900 (27.61)	1027 (30.64)	1121 (34.29)	1270 (38.58)	<0.001
Age, mean (SD)	T2DM	74.82 (10.87)	75.18 (10.95)	75.36 (11.15)	75.01 (11.16)	0.386
	No T2DM	68.45 (16.29)	68.76 (16.65)	69.03 (16.28)	68.33 (16.57)	0.041
18–49 years old, *n* (%)	T2DM	40 (2.01)	47 (2.14)	55 (2.07)	55 (2.2)	0.727
	No T2DM	891 (13.51)	990 (13.66)	965 (12.5)	1073 (13.57)	0.645
50–64 years old, *n* (%)	T2DM	315 (15.85)	316 (14.38)	395 (14.88)	387 (15.46)	0.920
	No T2DM	1512 (22.92)	1615 (22.28)	1749 (22.66)	1862 (23.55)	0.327
65–79 years old, *n* (%)	T2DM	859 (43.21)	940 (42.79)	1119 (42.16)	1090 (43.53)	0.932
	No T2DM	2241 (33.98)	2360 (32.55)	2624 (33.99)	2684 (33.95)	0.606
≥80 years old, *n* (%)	T2DM	774 (38.93)	894 (40.69)	1085 (40.88)	972 (38.82)	0.914
	No T2DM	1952 (29.59)	2285 (31.52)	2381 (30.85)	2287 (28.93)	0.275
CCI index, mean (SD)	T2DM	1.71 (1.11)	1.66 (1.12)	1.71 (1.11)	1.78 (1.14)	0.002
	No T2DM	1.31 (1.04)	1.34 (1.04)	1.35 (1.05)	1.37 (1.06)	0.011
LOHS, median (IQR)	T2DM	22 (24.5)	21 (23)	21(22)	23 (23.5)	0.135
	No T2DM	24 (29)	24 (29)	24 (28)	25 (30)	0.379
IHM, *n* (%)	T2DM	616 (30.99)	666 (30.31)	833 (31.39)	718 (28.67)	0.168
	No T2DM	1889 (28.64)	2075 (28.62)	2168 (28.09)	2182 (27.6)	0.438

HAP: hospital-acquired pneumonia; NV-HAP: non-ventilator hospital-acquired pneumonia; VAP: ventilator-associated pneumonia; T2DM: type 2 diabetes mellitus; CCI: Charlson comorbidity index; LOHS: length of hospital stay; IHM: in-hospital mortality.

**Table 2:** Distribution of study covariates and hospital outcomes of women with and without T2DM who developed hospital-acquired pneumonia (HAP) in Spain (2016–2019), before and after propensity score matching (PSM).

Variables	Before PSM			After PSM		
	T2DM	No T2DM	*p*-Value	T2DM	No T2DM	*p*-Value
*N* (incidence of HAP per 100,000 women hospitalized)	3185 (240.15)	10,247 (140.65)	<0.001			
*N* (incidence of NV-HAP per 100,000 women hospitalized)	2941 (221.75)	8986 (123.34)	<0.001			
*N* (incidence of VAP per 100,000 women hospitalized)	244 (18.39)	1261 (17.30)	0.382			
Age, mean (SD)	77.65 (11.17)	70.77 (17.06)	<0.001	77.65 (11.17)	78.8 (11.51)	<0.001
18–49 years old, *n* (%)	62 (1.95)	1296 (12.65)	<0.001	62 (1.95)	55 (1.73)	0.513
50–64 years old, *n* (%)	347 (10.89)	1991 (19.43)	<0.001	347 (10.89)	328 (10.3)	0.439
65–79 years old, *n* (%)	1144 (35.92)	3056 (29.82)	<0.001	1144 (35.92)	995 (31.24)	<0.001
≥80 years old, *n* (%)	1632 (51.24)	3904 (38.1)	<0.001	1632 (51.24)	1807 (56.73)	<0.001
CCI index, mean (SD)	1.55 (1.07)	1.22 (0.98)	<0.001	1.55 (1.07)	1.50 (1.04)	0.049
Myocardial infarction, *n* (%)	259 (8.13)	396 (3.86)	<0.001	259 (8.13)	211 (6.62)	0.021
Congestive heart failure, *n* (%)	1266 (39.75)	2643 (25.79)	<0.001	1266 (39.75)	1248 (39.18)	0.645
Peripheral vascular disease, *n* (%)	202 (6.34)	428 (4.18)	<0.001	202 (6.34)	170 (5.34)	0.087
Cerebrovascular disease, *n* (%)	567 (17.8)	1510 (14.74)	<0.001	567 (17.8)	532 (16.7)	0.246
Dementia, *n* (%)	275 (8.63)	601 (5.87)	<0.001	275 (8.63)	289 (9.07)	0.537
COPD, *n* (%)	493 (15.48)	1539 (15.02)	0.527	493 (15.48)	505 (15.86)	0.679
Rheumatoid disease, *n* (%)	93 (2.92)	366 (3.57)	0.077	93 (2.92)	140 (4.4)	0.002
Peptic ulcer, *n* (%)	48 (1.51)	193 (1.88)	0.162	48 (1.51)	51 (1.6)	0.761
Mild moderate/severe liver disease, *n* (%)	221 (6.94)	676 (6.6)	0.500	221 (6.94)	212 (6.66)	0.654
Hemiplegia or paraplegia, *n* (%)	165 (5.18)	608 (5.93)	0.111	165 (5.18)	136 (4.27)	0.087
Renal disease, *n* (%)	941 (29.54)	1376 (13.43)	<0.001	941 (29.54)	877 (27.54)	0.076
Cancer/Metastatic cancer, *n* (%)	407 (12.78)	2163 (21.11)	<0.001	407 (12.78)	398 (12.5)	0.734
AIDS, *n* (%)	6 (0.19)	51 (0.5)	0.019	6 (0.19)	8 (0.25)	0.593
Undergone surgery, *n* (%)	1165 (36.58)	4905 (47.87)	<0.001	1165 (36.58)	1305 (40.97)	<0.001
Bronchial fibroscopy, *n*(%)	48 (1.51)	274 (2.67)	<0.001	48 (1.51)	47 (1.48)	0.918
Computerized axial tomography of thorax, *n*(%)	203 (6.37)	694 (6.77)	0.431	203 (6.37)	208 (6.53)	0.799
Dialysis, *n*(%)	181 (5.68)	499 (4.87)	0.067	181 (5.68)	154 (4.84)	0.130
Oxygen prior to hospitalization *n* %	109 (3.42)	252 (2.46)	0.003	109 (3.42)	96 (3.01)	0.356
LOHS, Median (IQR)	21 (22)	23 (28)	<0.001	21 (22)	21 (23)	0.415
IHM, *n* (%)	968 (30.39)	2783 (27.16)	<0.001	968 (30.39)	960 (30.14)	0.827

HAP: hospital-acquired pneumonia; NV-HAP: non-ventilator hospital-acquired pneumonia; VAP: ventilator-associated pneumonia; T2DM: type 2 diabetes mellitus; CCI: Charlson comorbidity index; COPD: chronic obstructive pulmonary disease; AIDS: acquired immune deficiency syndrome; LOHS: length of hospital stay; IHM: in-hospital mortality.

**Table 3:** Distribution of study covariates and hospital outcomes of men with and without T2DM who developed hospital-acquired pneumonia (HAP) in Spain (2016–2019), before and after propensity score matching (PSM).

Variables	Before PSM			After PSM		
	T2DM	No T2DM	*p*-Value	T2DM	No T2DM	*p*-Value
*N* (incidence of HAP per 100,000 women hospitalized)	6158 (336.68)	19,224 (326.62)	0.038			
*N* (incidence of NV-HAP per 100,000 women hospitalized)	5494 (300.37)	16,167 (274.68)	<0.001			
*N* (incidence of VAP per 100,000 women hospitalized)	664 (36.30)	3057 (51.94)	<0.001			
Age, mean (SD)	73.8 (10.75)	67.51 (16.01)	<0.001	73.8 (10.75)	74.92 (11.23)	<0.001
18–49 years old, *n* (%)	135 (2.19)	2623 (13.64)	<0.001	135 (2.19)	132 (2.14)	0.853
50–64 years old, *n* (%)	1066 (17.31)	4747 (24.69)	<0.001	1066 (17.31)	1002 (16.27)	0.123
65–79 years old, *n* (%)	2864 (46.51)	6853 (35.65)	<0.001	2864 (46.51)	2615 (42.47)	<0.001
≥80 years old, *n* (%)	2093 (33.99)	5001 (26.01)	<0.001	2093 (33.99)	2409 (39.12)	<0.001
CCI index, mean (SD)	1.8 (1.14)	1.41 (1.08)	<0.001	1.8 (1.14)	1.75 (1.13)	0.009
Myocardial infarction, *n* (%)	845 (13.72)	1584 (8.24)	<0.001	845 (13.72)	788 (12.8)	0.130
Congestive heart failure, *n* (%)	1882 (30.56)	3751 (19.51)	<0.001	1882 (30.56)	1789 (29.05)	0.067
Peripheral vascular disease, *n* (%)	862 (14)	1794 (9.33)	<0.001	862 (14)	793 (12.88)	0.068
Cerebrovascular disease, *n* (%)	1092 (17.73)	2771 (14.41)	<0.001	1092 (17.73)	1093 (17.75)	0.981
Dementia, *n* (%)	299 (4.86)	721 (3.75)	<0.001	299 (4.86)	326 (5.29)	0.268
COPD, *n* (%)	1681 (27.3)	4536 (23.6)	<0.001	1681 (27.3)	1701 (27.62)	0.686
Rheumatoid disease, *n* (%)	95 (1.54)	243 (1.26)	0.097	95 (1.54)	101 (1.64)	0.666
Peptic ulcer, *n* (%)	124 (2.01)	424 (2.21)	0.367	124 (2.01)	139 (2.26)	0.350
Mild moderate/severe liver disease, *n* (%)	662 (10.75)	2090 (10.87)	0.789	662 (10.75)	627 (10.18)	0.303
Hemiplegia or paraplegia, *n* (%)	369 (5.99)	1210 (6.29)	0.393	369 (5.99)	338 (5.49)	0.230
Renal disease, *n* (%)	1745 (28.34)	2539 (13.21)	<0.001	1745 (28.34)	1593 (25.87)	0.002
Cancer/Metastatic cancer, *n* (%)	1414 (22.96)	5151 (26.79)	<0.001	1414 (22.96)	1445 (23.47)	0.508
AIDS, *n* (%)	19 (0.31)	213 (1.11)	<0.001	19 (0.31)	26 (0.42)	0.296
Undergone surgery, *n* (%)	2744 (44.56)	10,138 (52.74)	<0.001	2744 (44.56)	2966 (48.16)	<0.001
Bronchial fibroscopy, *n*(%)	145 (2.35)	670 (3.49)	<0.001	145 (2.35)	145 (2.35)	0.999
Computerized axial tomography of thorax, *n*(%)	428 (6.95)	1708 (8.88)	<0.001	428 (6.95)	515 (8.36)	0.003
Dialysis, *n*(%)	507 (8.23)	1358 (7.06)	0.002	507 (8.23)	477 (7.75)	0.319
Oxygen prior to hospitalization *n* %	212 (3.44)	490 (2.55)	<0.001	212 (3.44)	196 (3.18)	0.420
LOHS, Median (IQR)	22 (24)	25 (29)	<0.001	22 (24)	23 (26)	0.014
IHM, *n* (%)	1865 (30.29)	5531 (28.77)	0.023	1865 (30.29)	1938 (31.47)	0.155

HAP: hospital-acquired pneumonia; NV-HAP: non-ventilator hospital-acquired pneumonia; VAP: ventilator-associated pneumonia; T2DM: type 2 diabetes mellitus; CCI: Charlson comorbidity index; COPD: chronic obstructive pulmonary disease; AIDS: acquired immune deficiency syndrome; LOHS: length of hospital stay; IHM: in-hospital mortality.

**Table 4:** Distribution of study covariates and hospital outcomes of men and women with T2DM who developed hospital-acquired pneumonia (HAP) in Spain (2016–2019), before and after propensity score matching (PSM).

Variables	Before PSM			After PSM		
	T2DM Men	T2DM Women	*p*-Value	T2DM Men	T2DM Women	*p*-Value
*N* (incidence of HAP per 100,000 subjects hospitalized)	6158 (336.68)	3185 (240.15)	<0.001			
*N* (incidence of NV-HAP per 100,000 subjects hospitalized)	5494 (300.37)	2941 (221.75)	<0.001			
*N* (incidence of VAP per 100,000 subjects hospitalized)	664 (36.30)	244 (18.39)	<0.001			
Age, mean (SD)	73.8 (10.75)	77.65 (11.17)	<0.001	77.88 (9.20)	77.65 (11.17)	0.375
18–49 years old, *n* (%)	135 (2.19)	62 (1.95)	0.433	5 (0.16)	62 (1.95)	<0.001
50–64 years old, *n* (%)	1066 (17.31)	347 (10.89)	<0.001	296 (9.29)	347 (10.89)	0.034
65–79 years old, *n* (%)	2864 (46.51)	1144 (35.92)	<0.001	1317 (41.35)	1144 (35.92)	<0.001
≥80 years old, *n* (%)	2093 (33.99)	1632 (51.24)	<0.001	1567 (49.2)	1632 (51.24)	0.103
CCI index, mean (SD)	1.8 (1.14)	1.55 (1.07)	0.049	1.45 (1.05)	1.55 (1.07)	0.176
Myocardial infarction, *n* (%)	845 (13.72)	259 (8.13)	<0.001	222 (6.97)	259 (8.13)	0.079
Congestive heart failure, *n* (%)	1882 (30.56)	1266 (39.75)	<0.001	1266 (39.75)	1266 (39.75)	0.999
Peripheral vascular disease, *n* (%)	862 (14)	202 (6.34)	<0.001	105 (3.3)	202 (6.34)	<0.001
Cerebrovascular disease, *n* (%)	1092 (17.73)	567 (17.8)	0.934	604 (18.96)	567 (17.8)	0.231
Dementia, *n* (%)	299 (4.86)	275 (8.63)	<0.001	263 (8.26)	275 (8.63)	0.589
COPD, *n* (%)	1681 (27.3)	493 (15.48)	<0.001	402 (12.62)	493 (15.48)	0.001
Rheumatoid disease, *n* (%)	95 (1.54)	93 (2.92)	<0.001	62 (1.95)	93 (2.92)	0.012
Peptic ulcer, *n* (%)	124 (2.01)	48 (1.51)	0.084	72 (2.26)	48 (1.51)	0.027
Mild moderate/severe liver disease, *n* (%)	662 (10.75)	221 (6.94)	<0.001	183 (5.75)	221 (6.94)	0.051
Hemiplegia or paraplegia, *n* (%)	369 (5.99)	165 (5.18)	0.109	178 (5.59)	165 (5.18)	0.471
Renal disease, *n* (%)	1745 (28.34)	941 (29.54)	0.222	955 (29.98)	941 (29.54)	0.701
Cancer/Metastatic cancer, *n* (%)	1414 (22.96)	407 (12.78)	<0.001	285 (8.95)	407 (12.78)	<0.001
AIDS, *n* (%)	19 (0.31)	6 (0.19)	0.287	6 (0.19)	6 (0.19)	0.999
Undergone surgery, *n* (%)	2744 (44.56)	1165 (36.58)	<0.001	1205 (37.83)	1165 (36.58)	0.300
Bronchial fibroscopy, *n* (%)	145 (2.35)	48 (1.51)	0.006	43 (1.35)	48 (1.51)	0.598
Computerized axial tomography of thorax, *n* (%)	428 (6.95)	203 (6.37)	0.292	180 (5.65)	203 (6.37)	0.225
Dialysis, *n* (%)	507 (8.23)	181 (5.68)	<0.001	213 (6.69)	181 (5.68)	0.096
Oxygen prior to hospitalization *n* %	212 (3.44)	109 (3.42)	0.959	92 (2.89)	109 (3.42)	0.223
LOHS, Median (IQR)	22 (24)	21 (22)	0.415	21 (23)	21 (22)	<0.001
IHM, *n* (%)	1865 (30.29)	968 (30.39)	0.915	976 (30.64)	968 (30.39)	0.828

HAP: hospital-acquired pneumonia; NV-HAP: non-ventilator hospital-acquired pneumonia; VAP: ventilator-associated pneumonia; T2DM: type 2 diabetes mellitus; CCI: Charlson comorbidity index; COPD: chronic obstructive pulmonary disease; AIDS: acquired immune deficiency syndrome; LOHS: length of hospital stay; IHM: in-hospital mortality.

**Table 5:** Multivariable analysis of factors associated with in-hospital mortality who developed hospital-acquired pneumonia (HAP) among T2DM patients according to sex.

Variables	Men	Women	Both
	OR (95% CI)	OR (95% CI)	OR (95% CI)
18–49 years old	1	1	1
50–64 years old	1.46 (0.97–2.64)	1.74 (0.87–3.49)	1.86 (0.95–3.62)
65–79 years old	1.54 (1.02–2.33)	1.73 (0.89–3.39)	2.04 (1.07–3.92)
≥80 years old	1.87 (1.23–2.84)	2.08 (1.06–4.08)	2.42 (1.26–4.66)
Myocardial infarction	1.22 (1.04–1.43)	1.35 (1.03–1.78)	1.36 (1.12–1.66)
Congestive heart failure	1.15 (1.02–1.3)	1.36 (1.15–1.6)	1.23 (1.09–1.38)
Peripheral vascular disease			1.38 (1.08–1.77)
Cerebrovascular disease	1.54 (1.33–1.77)	1.58 (1.3–1.93)	1.62 (1.41–1.86)
Dementia	1.29 (1.01–1.67)		1.24 (1.02–1.5)
Mild moderate/severe liver disease	1.28 (1.06–1.53)		1.33 (1.07–1.65)
Cancer/Metastatic cancer	1.51 (1.32–1.73)	1.86 (1.48–2.34)	1.7 (1.43–2.02)
Undergone surgery	0.81 (0.72–0.92)	0.74 (0.62–0.88)	0.8 (0.71–0.91)
Bronchial fibroscopy	1.4 (0.99–1.99)	1.75 (1.1–3.22)	2.01 (1.31–3.11)
Dialysis	2.62 (2.16–3.19)	2.03 (1.47–2.8)	2.15 (1.72–2.67)
2017	1.05 (0.89–1.24)	0.91 (0.72–1.15)	1 (0.85–1.17)
2018	1.08 (0.92–1.26)	0.9 (0.73–1.12)	1 (0.85–1.16)
2019	0.9 (0.77–1.06)	0.8 (0.64–1)	0.84 (0.72–0.98)
VAP	2.11 (1.76–2.52)	2.36 (1.76–3.16)	2.09 (1.7–2.57)
Male sex			1.03 (0.92–1.15)

## Data Availability

According to the contract signed with the Spanish Ministry of Health and Social Services, which provided access to the databases from the Spanish National Hospital Database (Conjunto Mínimo Basico de Datos; CMBD), we cannot share the databases with any other investigator, and we have to destroy the databases once the investigation has concluded. Consequently, we cannot upload the databases to any public repository. However, any investigator can apply for access to the databases by filling out the questionnaire available at http://www.msssi.gob.es/estadEstudios/estadisticas/estadisticas/estMinisterio/SolicitudCMBDdocs/Formulario_Peticion_Datos_CMBD.pdf (accessed on 6 September 2021). All other relevant data are included in the paper.

## Supplementary Materials

The following are available online at https://www.mdpi.com/article/10.3390/ijerph182312645/s1, Table S1: ICD-10 codes for diagnosis and therapeutic procedures used in this investigation, Table S2: Distribution of pneumonia pathogens in patients with and without T2DM who developed hospital-acquired pneumonia (HAP) in Spain from 2016 to 2019. Table S3: Distribution of pneumonia pathogens in women and men with T2DM who developed hospital-acquired pneumonia (HAP), in Spain (2016–19), before and after propensity score matching.
