# Supplementary material for: Sex Differences in Hospital-Acquired Pneumonia among Patients with Type 2 Diabetes Mellitus Patients: Retrospective Cohort Study using Hospital Discharge Data in Spain (2016–2019)
Source: Int J Environ Res Public Health. 2021 Nov 30;18(23):12645. doi: 10.3390/ijerph182312645 (PMC8656727; doi:10.3390/ijerph182312645)
Supplement: Supplementary file 1 [file ijerph-18-12645-s001.zip › ijerph-1393835-supplementary.pdf]

Table S1. ICD-10 codes for diagnosis and therapeutic procedures used in this investigation

		ICD-10 codes
Hospital acquired pneumonia	Non ventilator hospital-acquired pneumonia (NV-HAP)*	J12 to J18 in any diagnosis fields (2-20) and with a POA indicator coded as "N" who had a hospitalization ≥48 hours
	Ventilator-associated pneumonia (VAP)*	J95.851 in any diagnosis fields (2-20) and a POA indicator coded as "N"
Bronchial fibroscopy		0BJ08ZZ
Computerized axial tomography of thorax		BW24
Dyalisis		5A1D00Z, 5A1D60Z,3E1M39Z
Oxygen prior to hospitalization		Z99.81
Aspergillus		B44.9
Candidiasis		B37.1
Escherichia coli		J15.5
Haemophilus influenzae		J14
Klebsiella pneumoniae		J15
Legionella		A48.1
Non specified *Streptococcus*		J15.4
Other Gram negative bacteria		J15.6
Influenza virus		J09.X1, J10.00 J10.01, J10.08, J11.0, J11.00, J11.08
Other virus		J12.XX
Pseudomonas aeruginosa		J15.1
Staphylococcus aureus		J15.211 AND J15.212
Streptococcus pneumoniae		J13

*Each discharge diagnosis has a "Present on Admission (POA)" indicator assigned according to the ICD-10-CM Official Guidelines for Coding and Reporting (**https://icdlist.com/icd-10/guidelines/**). The reporting options and definitions for POA are "Y" (present at admission); "N" (not present at admission); "U" (lack documentation to determine presence at admission); "W" (provider is unable to clinically determine if the condition was present); and unreported/not used.

Table S2. Distribution of pneumonia pathogens in patients with and without T2DM who developed hospital-acquired pneumonia (HAP) in Spain from 2016 to 2019

		2016	2017	2018	2019	p-value
Aspergillus, n(%)	**T2DM**	4(0.2)	2(0.09)	6(0.23)	4(0.16)	0.701
	**No T2DM**	25(0.38)	24(0.33)	15(0.19)	26(0.33)	0.203
Candidiasis, n(%)	**T2DM**	14(0.7)	16(0.73)	19(0.72)	15(0.6)	0.944
	**No T2DM**	47(0.71)	54(0.74)	53(0.69)	74(0.94)	0.275
Escherichia coli, n(%)	**T2DM**	33(1.66)	25(1.14)	43(1.62)	26(1.04)	0.144
	**No T2DM**	139(2.11)	122(1.68)	135(1.75)	131(1.66)	0.163
Haemophilus influenzae, n(%)	**T2DM**	13(0.65)	19(0.86)	32(1.21)	26(1.04)	0.261
	**No T2DM**	104(1.58)	89(1.23)	109(1.41)	102(1.29)	0.302
Klebsiella pneumoniae, n(%)	**T2DM**	57(2.87)	45(2.05)	58(2.19)	74(2.96)	0.105
	**No T2DM**	202(3.06)	204(2.81)	209(2.71)	249(3.15)	0.330
Legionella, n(%)	**T2DM**	0(0)	1(0.05)	1(0.04)	3(0.12)	0.351
	**No T2DM**	4(0.06)	4(0.06)	7(0.09)	7(0.09)	0.798
Non specified *Streptococcus*, n(%)	**T2DM**	8(0.4)	4(0.18)	5(0.19)	7(0.28)	0.447
	**No T2DM**	42(0.64)	25(0.34)	34(0.44)	42(0.53)	0.080
Other Gram-negative bacteria, n(%)	**T2DM**	50(2.52)	59(2.69)	59(2.22)	71(2.84)	0.547
	**No T2DM**	214(3.24)	242(3.34)	244(3.16)	255(3.23)	0.944
Pseudomonas aeruginosa, n(%)	**T2DM**	65(3.27)	83(3.78)	76(2.86)	89(3.55)	0.315
	**No T2DM**	344(5.22)	338(4.66)	338(4.38)	357(4.52)	0.098
Streptococcus pneumoniae, n(%)	**T2DM**	41(2.06)	48(2.18)	66(2.49)	57(2.28)	0.796
	**No T2DM**	140(2.12)	177(2.44)	223(2.89)	238(3.01)	0.003
Staphylococcus aureus, n(%)	**T2DM**	26(1.31)	52(2.37)	55(2.07)	58(2.32)	0.059
	**No T2DM**	208(3.15)	236(3.26)	262(3.39)	265(3.35)	0.857
Influenza virus, n(%)	**T2DM**	15(0.75)	18(0.82)	41(1.54)	27(1.08)	0.033
	**No T2DM**	90(1.36)	46(0.63)	117(1.52)	106(1.34)	<0.001
Other virus, n (%)	**T2DM**	13(0.65)	10(0.46)	11(0.41)	18(0.72)	0.406
	**No T2DM**	34(0.52)	39(0.54)	64(0.83)	64(0.81)	0.026

T2DM: Type 2 diabetes mellitus;

Table S3. Distribution of pneumonia pathogens in women and men with T2DM who developed hospital-acquired pneumonia (HAP), in Spain (2016-19), before and after propensity score matching.

	Before PSM			After PSM		
	**T2DM Men**	**T2DM Women**	**p-value**	**T2DM Men**	**T2DM Women**	**p-value**
Aspergillus, n(%)	7(0.11)	9(0.28)	0.061	3(0.09)	9(0.28)	0.083
Candidiasis, n(%)	46(0.75)	18(0.57)	0.312	17(0.53)	18(0.57)	0.865
Escherichia coli, n(%)	102(1.66)	25(0.78)	0.001	52(1.63)	25(0.78)	0.002
Haemophilus influenzae, n(%)	67(1.09)	23(0.72)	0.086	32(1)	23(0.72)	0.223
Klebsiella pneumoniae, n(%)	181(2.94)	53(1.66)	<0.001	78(2.45)	53(1.66)	0.027
Legionella, n(%)	5(0.08)	0(0)	0.108	3(0.09)	0(0)	0.083
Non specified *Streptococcus*, n(%)	21(0.34)	3(0.09)	0.025	11(0.35)	3(0.09)	0.032
Other Gram-negative bacteria, n(%)	201(3.26)	38(1.19)	<0.001	105(3.3)	38(1.19)	<0.001
Pseudomonas aeruginosa, n(%)	228(3.7)	85(2.67)	0.008	92(2.89)	85(2.67)	0.594
Streptococcus pneumoniae, n(%)	155(2.52)	57(1.79)	0.025	65(2.04)	57(1.79)	0.465
Staphylococcus aureus, n(%)	131(2.13)	60(1.88)	0.430	71(2.23)	60(1.88)	0.331
Influenza viruse, n(%)	58(0.94)	43(1.35)	0.071	30(0.94)	43(1.35)	0.126
Other virus, n(%)	31(0.5)	21(0.66)	0.337	11(0.35)	21(0.66)	0.076
